# Metacognitive training for psychosis (MCT): past, present, and future

**DOI:** 10.1007/s00406-022-01394-9

**Published:** 2022-03-25

**Authors:** Steffen Moritz, Mahesh Menon, Ryan Balzan, Todd S. Woodward

**Affiliations:** 1 Department of Psychiatry and Psychotherapy, University Medical Center Hamburg-Eppendorf, Hamburg, Germany; 2 Department of Psychiatry, University of British Columbia, Vancouver, BC Canada; 3 Mental Health and Addictions Research Institute, Vancouver, BC Canada; 4 College of Education, Psychology and Social Work, Flinders University, Bedford Park, SA Australia; 5 Orama Institute, Flinders University, Bedford Park, SA Australia; 6 BC Psychosis Program, Vancouver Coastal Health, Vancouver, BC Canada

**Keywords:** Schizophrenia, Psychosis, Cognitive biases, Metacognitive training, Social cognition

## Abstract

This article provides an overview and retrospective on metacognitive training for psychosis (MCT), which first appeared approximately 2 decades ago. We recount how our empirical understanding of psychosis at that time led to the first preliminary version of the program. We describe setbacks and challenges that led to major changes, including revisions to existing modules (e.g., more focus on metacognitive variables, particularly on decision confidence as one of the primary targets of treatment) and the creation of new modules addressing mood, as well as attempts to improve sustainability of effects via homework exercises and a smartphone app (www.uke.de/mct_app). We have also enhanced dissemination efforts by creating new culturally sensitive language versions and facilitating low-threshold training through e-learning courses (www.uke.de/e-mct). Finally, we discuss several meta-analyses on the efficacy of MCT that have been published over the last decade. While reviews were initially inconsistent, possibly reflecting the insufficient statistical power and lower design quality of the first MCT studies, more recent meta-analyses have confirmed the efficacy of MCT on positive symptoms, insight, and cognitive biases, which has led to the inclusion of MCT in some national treatment guidelines for schizophrenia.

## Metacognitive training for psychosis: the beginning

The origin of the idea to develop an intervention focused on cognitive distortions in people with psychosis emerged in 2001 when the first author, Dr. Steffen Moritz, visited the experimental laboratory of Dr. Peter Graf at the Department of Psychology at the University of British Columbia (UBC; Vancouver, Canada) as a postdoctoral fellow. There he met the senior author, Dr. Todd Woodward, also a postdoctoral fellow in the laboratory at the time. In the years that followed, they were joined in their shared research endeavors by Drs. Mahesh Menon and Ryan Balzan, who also held postdoctoral positions at UBC (in 2004 and 2012). In our early discussions, we shared our experience that most neurocognitive research in psychosis lacked face validity in terms of its connections to the core symptoms of psychosis (e.g., we could not imagine how problems with memory and attention could explain specific positive symptoms) and that cognitive variables were rather poorly correlated with symptom severity [1] and lacked specificity for the disorder [for a recent meta-analysis, see [2]]. While the findings of neurocognitive deficits, such as difficulties with verbal memory and executive functions, are highly relevant to our understanding of functional outcome, their relationship to positive symptoms in schizophrenia—our primary area of interest and the conventional target in treatment for schizophrenia—remains low.

At that time, a small but growing body of work was concerned with cognitive biases and their relationship to paranoia, which was summarized nicely in a 1999 review by Garety and Freeman [3]. Biases, unlike impairments, are ubiquitous and also exist within the nonclinical population [4]; cognitive biases are distortions in the way we collect, process, remember, and evaluate information. In fact, some biases, such as the self-serving bias and unrealistic optimism, may even promote subjective well-being [5].

This line of research, which stood on the shoulders of the seminal work by psychologists Tversky and Kahneman [6], intrigued us. The “jumping to conclusions” bias was already thought to be a driving factor in the development of delusions [7]. One key theme in this new line of research was that cognitive biases can occur with delusion-neutral content, suggesting that biases drive the delusional process rather than being epiphenomena of the delusions themselves (i.e., jumping to conclusions due to paranoid thinking). As we considered which factors might play a role in the maintenance of delusions, we started to examine how individuals with delusions look at evidence for their beliefs and the mechanisms that lead them to stick to their implausible interpretations of reality. These studies led to the discovery of other biases that may serve as maintenance factors, including the bias against disconfirmatory evidence (BADE) [8], liberal acceptance (a post hoc explanation of results we obtained using a thematic apperception test) [9], and the “overconfidence in errors” bias [10]. The latter was inspired by a highly schizotypal person the first author met at that time. This person told fantastical stories with utter conviction but with no indication of awareness of confabulation or fabricated memories, raising the question of whether the overconfidence in false memories bias may be independent of symptom phase and may manifest beyond delusional content [10].

At this point, our investigations were entirely devoted to basic research. In 2002, the former mentor of the first author and the director of the Department of Psychiatry at the University Medical Center Hamburg, Prof. Dieter Naber, urged the first author to quit the “ivory tower of basic research” and run a cognitive training group based on these emerging findings. The idea emerged to develop a program that would help patients understand the manifestation of cognitive biases in their lives, with the hope that this awareness might increase their insight and reduce their delusions. Within several weeks, we developed a pilot program consisting of eight modules that are very similar to how MCT still looks today. Groups were run twice weekly in the outpatient ward for schizophrenia in Hamburg. In these early days, we were sometimes just 1 week ahead of module development. To save time, we used the same stimuli and materials we had used in our basic research, and we also asked colleagues for permission to use some of the experimental stimuli from their published studies. This interim solution became somewhat permanent. For example, the material from Module 1 on the attribution bias is largely based on the Internal, Personal and Situational Attributions Questionnaire (IPSAQ) [11]. Module 2 on the jumping to conclusions bias was derived from studies that we later published in 2006 [12]. Module 3 on belief inflexibility and changing beliefs was borrowed from the materials we used for a BADE task published in 2006 [13]. Table 1 summarizes the common challenges encountered in psychotherapy for psychosis and how we addressed these over the years.

**Table 1:** Challenges to psychotherapeutic interventions for psychosis and how MCT addresses them

Challenges to psychotherapy for people with psychosis	How these challenges are addressed in MCT
Long and expensive training and materials for clinicians	Free download of MCT materials and manual, donation-based e-learning, certification encouraged but not mandatory to conduct sessions with patients
Lack of availability of psychologists specialized in psychosis	While the MCT is perhaps best carried out by psychologists or psychiatrists, occupational therapists and psychiatric nurses with special training in psychiatry are also deemed competent to facilitate MCT. A short and easily understood manual and e-learning (www.uke.de/e-mct) ensure that even nonacademic clinicians and psychology students at the bachelor’s or master’s level may facilitate the intervention
Closed groups often “dry out,” and new patients are not offered treatment in the interim phase or once a closed group has started	The open group concept allows rolling intake; the independent modules do not build upon each other
Lack of motivation and adherence	MCT has playful, fun exercises with content that neither challenges personal delusions nor stigmatizes psychosis; in addition, gamification elements have been integrated into the app (COGITO; www.uke.de/mct_app)
Maintenance of psychotherapy can be low; longer-term follow-up results are usually worse than immediate post-therapy effects [49]	Conventional homework sheets that many patients chose not to read or work on are now complemented by the transdiagnostic app that sends daily reminders and is available in many languages. The app offers exercises from MCT, mindfulness, CBT, and acceptance and commitment therapy (ACT) Since many participants have a poor attention span for reasons such as medication side effects and primary neurocognitive deficits, we have developed a parallel cycle for most language versions to ensure that the learning aims are internalized Inspired by a training program for soldiers with PTSD who often have anger management problems, we developed a “yellow card” that the patient can carry with them that summarizes the core learnings of the training and provides helpful reminders during stressful moments or crises (the “red card” allows the patient to note the names and phone numbers of key persons the patient trusts)
Cultural adaptations/personalization	Early on, we asked translators to adapt the MCT modules to their cultures. Yet, we continued to get requests for greater consideration of culture-specific issues. To meet this aim, we decided to provide an open source version of MCT that allows clinicians to adapt the PowerPoint material and create their own MCT stimuli. We wanted to allow experienced clinicians to incorporate their views and experiences into the training while being true to the rationale and basic tenets of MCT

In the early iterations of MCT groups, we worried that the patients would feel insulted or stigmatized or become defensive if we explicitly stated that certain cognitive biases were linked to psychosis, so we did not elaborate on this association. When a participant during a session once literally asked “Why are we doing this?” we rethought our position and decided to address this exact question in every module. This fully transparent approach has since become a hallmark feature of MCT, and, according to our participants, they have appreciated this transparency. We also feel that the notion that all people experience cognitive bias, individuals with and without psychosis, normalizes the experience and is less stigmatizing than the concept of neurocognitive impairment (thus, we provide many examples of how most people are prone to biases and mistakes). Over time, we also expanded sections to ensure that each bias was framed as relevant to daily life by offering a range of experiential “games” and short case studies as we realized that some patients had difficulty connecting the themes and exercises to their daily lives.

In the early versions of the intervention, we aimed to keep the focus of MCT on cognitive biases associated with delusions and did not consider the work on biases seen in depression, which are typically addressed in cognitive behavior therapy (CBT). However, no CBT group was available on the ward where we were administering MCT. Thus, to fill this gap, we created what was then the final module, which focused on challenging depressive cognitions and low self-esteem (module 8). Initially, we felt that this module was an outlier and somewhat muddied the waters of the central “cognitive bias” theme. Yet, since low self-esteem and depression were addressed in the review by Garety and Freeman [3], we felt that incorporating at least one emotion-focused module could be justified. As conceptualizations of CBT for psychosis developed, we realized that depression-related biases and low self-esteem could play a role in maintaining delusions; indeed, many studies have shown that social isolation, avoidance, and self-blame may fuel positive symptoms [14]. These ideas led us to subsequently expand on these topics and to add specific modules on self-esteem and the impacts of stigma.

We had chosen the term “metacognitive training” as a working title as it captures one of several important features of metacognition—the correspondence of subjective and objective cognition—as we suspected that patients had little (cognitive) insight into their own biases [15–17]. In addition, confidence is regarded as a core aspect of metacognition in experimental psychology [18], and patients liked the term “metacognition.” We did not realize at the time that another metacognitive intervention, “metacognitive therapy,” was being developed by Adrian Wells and colleagues, creating some confusion that persists even today. This also led to some debate around what constitutes “cognitive” versus “metacognitive” levels of processing [19], and we have now published a number of articles where we highlight the differences as well as the shared cognitive and metacognitive features of metacognitive training, metacognitive therapy, metacognitive reflection and insight therapy (MERIT), and CBT (in fact, the “mantra” of CBT that “thoughts are thoughts and not facts” is itself a metacognitive statement) [20, 21].

## The future of MCT

Even experienced clinicians are often reluctant to engage in therapy with patients with schizophrenia, partly owing to lingering concerns regarding whether psychotherapy is appropriate, treatment nihilism, and concerns over poor adherence as well as poor therapeutic alliance. Despite the low-threshold approach of MCT, with its free materials, highly structured sessions, and a short comprehensive manual that makes the administration of the training rather self-explanatory, achieving wide dissemination was challenging. We increasingly were asked to provide workshops, which posed problems in terms of staffing (both for us and for clinicians who had to find time to attend the workshops). In 2020, we established a donation-based e-learning curriculum (www.uke.de/e-mct) that lasts approximately 10 h and ends with a certified exam (10 continuing education [CE] credits are awarded to therapists; earning CE points is an annual requirement for psychotherapists in Germany). The training is available in German and English, and so far more than 3000 clinicians from diverse clinical backgrounds (e.g., psychiatrists, psychologists, occupational therapists, nurses) have completed the training. We aim to expand these courses to other languages. During the COVID-19 pandemic, a number of clinician collaborators have also begun to deliver MCT remotely (using Zoom or other software), allowing patients living far from treatment centres to access the groups [22]. The efficacy of the remote delivery of MCT is now being formally assessed in randomized controlled trials.

Recently, we also developed an app to complement MCT. This app, which we initially called MCT and More, has been renamed COGITO (www.uke.de/cogito_app). Its goal is to improve adherence to and maintenance of treatment gains. We had noticed that many patients were not doing the homework due to forgetfulness, disorganization, lack of motivation, or lack of insight. Patients are now sent daily notifications via a smartphone app, which (when clicked by the patient) opens short exercises borrowed from CBT as well as third wave approaches, including MCT. The patient can choose one or two times during the day to receive these notifications. The app can also be personalized. Exercises that a user finds useful are shown more often if they press the favorite button. The user may also disable or edit exercises and can even upload their own exercises, which can be accompanied by photos. In the new version, famous quotes are displayed to promote self-reflection, hope, and better self-esteem. Two studies on populations with psychological problems (not necessarily schizophrenia) have shown that the app is effective as a stand-alone technique [23, 24]. We aim to expand COGITO and add new exercises as well as further features (e.g., audio readings of the exercises, simpler language), with the goal of creating material that will engage individuals who may have cognitive challenges.

We also believe that issues related to relationships (both friendships and romantic relationships) are crucial to recovery, and we are working on updating materials in the modules on theory of mind and depression/self-esteem to include such issues. As mentioned, for some years MCT has been available as an open source intervention, and we invite clinicians to continue to update and add material to the existing modules.

There is a burgeoning literature on thinking biases popularized by books such as The Black Swan [25] and Thinking, Fast and Slow [26]. It is doubtful that MCT covers all biases relevant to schizophrenia, and researchers have already begun to integrate other biases, such as illusory control [27]. Moreover, not all patients show the biases covered in MCT, and for some domains, particularly theory of mind, evidence for an association with delusions remains inconsistent. As early as 1999, Garety and Freeman [3] had questioned whether theory of mind deficits are specific to delusions or even to schizophrenia. Yet, we chose to incorporate this aspect because deficits in social cognition likely fuel problems with social interactions and may thus may be important in delusional ideation.

## Empirical evidence

Initially, the evidence for MCT was mixed. The first meta-analysis [28] considered only four studies and found a significant effect on the Positive and Negative Symptom Scale (PANSS) positive but not on the Psychotic Symptom Rating Scales (PSYRATS). Likewise, the meta-analysis by van Osterhout et al. was largely negative, although the authors acknowledged in a subsequent commentary [29] that when newer studies were considered alongside the older studies in the meta-analysis, the effect of MCT on positive symptoms (*g* = 0.32) and delusions (*g* = 0.31) were significant. The results of that meta-analysis were heavily influenced by a large-sample study from their own group [30], which found no effect of MCT in those with more severe delusions relative to a control group. That study was carefully designed and the protocol had been discussed with and approved by the first author; therefore, the null results gave us pause, and we reflected on and discussed ways we could improve the efficacy of MCT.

As a result, we first refined the recommended inclusion criteria for the MCT group to patients with mild to moderate delusions and suggested excluding individuals with severe delusions who were unwilling to engage in discussions as well as individuals with significant formal thought disorder, hostility, or antisocial behavior, as these are contraindicated for group therapy. The trial by van Oosterhout was conducted with many patients who were experiencing an acute exacerbation of psychosis, which might have compromised adherence and group cohesion. For these patients, we now recommend individualized treatment [31, 32] and, more recently, the briefer adaptation known as MCT acute (see www.uke.de/mct-acute; available since 2020). While early versions of MCT placed a particular emphasis on the jumping to conclusions and belief inflexibility biases, we refined the focus of the sessions to emphasize the importance of reducing overconfidence and stressed the importance of utilizing and generalizing the strategies outside the group (through homework, the “yellow card” and the “red card,” and the app; see Table 1). Indeed, it seems that a reduction in response confidence combined with an increased willingness to consider alternatives is a pivotal mechanism of improvement [33] and perhaps a recovery factor shared with antipsychotics [34].

Subsequent meta-analyses on MCT have concluded that MCT is effective in reducing positive symptoms and delusions [35]. Eichner and Berna’s meta-analysis [36] found significant effects for positive symptoms (delusions) and large effects for (subjective) acceptance, suggesting that the training was well tolerated by participants [for similar findings on symptoms, see 35]. Moreover, a meta-analysis from researchers based in Taiwan [37] found that MCT effects were sustained over time. Sauvé et al. [38] reported that metacognitive interventions were effective for cognitive biases, positive symptoms, and insight. The positive effects on insight have recently been corroborated by Lopez-Morinigo and coworkers [39]. A recent meta-analysis by Penney and colleagues [40] found a medium effect for positive symptoms and a medium-to-large effect for delusions. Stronger effects emerged because of new controlled studies with large effects that had not been included in the earlier meta-analyses. The authors also found a significant effect for self-esteem, negative symptoms, and functioning. Yet, the effect for negative symptoms was small, so for this syndrome we recommend an adapted version of negative symptoms by Linda Swanson (see www.uke.de/mct).

So far, no meta-analysis has investigated whether the expanded 10-module version of MCT, which has modules on self-esteem and (self-)stigma, is superior to the standard MCT with its eight modules. We decided to add these two complementary modules to MCT because patients wished to address emotional problems in treatment (in addition to positive symptoms such as delusions and hallucinations, the conventional target of schizophrenia treatment) [41–43]. These modules are also important as low mood and self-esteem can impact the symptoms of psychosis directly (e.g., the content of the hallucinations) and indirectly (as social isolation can exacerbate paranoia) [44]. Discussing mood can also be easier as most patients with psychosis have better awareness of and insight into their depressive symptoms than their psychosis. Patients may also prefer to discuss these issues as they often have ambivalence towards their positive symptoms. Importantly, fear and depression (especially in “bad me” paranoia) on the one hand and elation and feelings of grandeur on the other hand as many paranoid thoughts (e.g., being spied on by the Secret Service) may provide the individual with a sense of power and importance [45].

The expanded modules were first tested in a Japanese population [46]. The authors found significant effects not only on positive symptoms but also on functioning. Favorable effects on the expanded version were also detected in a recent study in China across all symptom domains, including negative symptoms and quality of life [47]. Using the expanded version in Chile [48], effects were found for positive symptoms and for the total effect (personal communication). Whether the additional modules and the app augment the established effects of the MCT has not been directly demonstrated; it is a question for future research.

## Conclusion

There is strong evidence from a number of meta-analyses that MCT improves positive symptoms, and there is also preliminary evidence suggesting that newer versions addressing self-esteem and (self-)stigma effectively improve other symptom domains. While the effect sizes appear to be in the range of other treatments for the signs and symptoms of schizophrenia—such as CBT and cognitive remediation, which overlap with MCT—the current effect sizes could be further improved in various ways. Whether the app, more sessions (e.g., parallel cycles), or complementing cognitive bias modification with work on emotional problems can help increase effect size remains to be demonstrated. Clearly, we should aim to achieve the effect sizes seen in CBT for depression and anxiety, given the chronic and debilitating symptoms of psychosis, to translate the effects of MCT into meaningful changes in everyday life and outcomes that improve patients’ lives. Online training may also be a fruitful approach that could reach larger numbers of patients.
